# The expanding family of regulatory B cells

**DOI:** 10.1093/intimm/dxv038

**Published:** 2015-06-12

**Authors:** Claudia Mauri, Madhvi Menon

**Affiliations:** Centre for Rheumatology, Division of Medicine, University College London, 5 University Street, London WC1E 6JF, UK

**Keywords:** B cell, Bregs, human, IL-10, immune-suppression, mouse, regulation, regulatory B cells, subsets

## Abstract

Over the last decade it has become evident that in addition to producing antibody, B cells activate the immune system by producing cytokines and via antigen presentation. In addition, B cells also exhibit immunosuppressive functions via diverse regulatory mechanisms. This subset of B cells, known as regulatory B cells (Bregs), contributes to the maintenance of tolerance, primarily via the production of IL-10. Studies in experimental animal models, as well as in patients with autoimmune diseases, have identified multiple Breg subsets exhibiting diverse mechanisms of immune suppression. In this review, we describe the different Breg subsets identified in mice and humans, and their diverse mechanisms of suppression in different disease settings.

## Abstract (precis)

The expanding family of regulatory B cells

## Introduction

Over the last decade, the role of regulatory B cells (Bregs) in suppressing pathological immune responses has been widely recognized (1). Bregs, as negative regulators of the immune system, prevent potentially damaging autoimmune and protective immune responses that can result in uncontrolled inflammation. The number of Bregs is therefore a crucial determinant of how the immune system is regulated; too few Bregs can result in autoimmunity and other inflammatory disorders, whereas too many Bregs can cause immune suppression.

Multiple studies in both mice and humans have demonstrated that Bregs suppress inflammatory responses primarily via the provision of IL-10 (1). Thus, IL-10 is not just a marker for Breg identification but also an important mediator of immune suppression by Bregs. With the increased interest in Breg biology in recent years, many new subsets of Bregs and additional mechanisms of Breg-mediated suppression have been identified. In this review, we explore the recent advances in the phenotypic and functional diversity of Bregs, in both mice and humans. We also highlight the diseases associated with defects in Breg homeostasis.

## Mouse Breg subsets

The evidence that B cells play an immune-regulatory role was first demonstrated in B-cell-deficient (µMT) mice that were unable to recover from experimental autoimmune encephalitis (EAE) (2). A few years later, different approaches led to the identification that it was a specific subset of B cells, namely, those producing IL-10 that exerted this suppressive function. Chimeric mice with IL-10 deficiency restricted to B cells displayed a persistent pro-inflammatory T_h_1 response and did not recover from EAE (3).

Mizoguchi *et al*., who coined the term ‘regulatory B cells’, reported the existence of a discrete population of IL-10-producing CD1d^hi^ Bregs that expanded with progression of chronic intestinal inflammatory conditions. CD1d^hi^ Bregs inhibited disease progression by down-regulating the inflammatory cascades associated with IL-1 and STAT3 activation (4). Simultaneously, our research group demonstrated that CD40-mediated activation of splenocytes induced the differentiation of IL-10-producing B cells that upon adoptive transfer suppress the arthritogenic T_h_1 response and the severity of collagen-induced arthritis (CIA) (5). The importance of endogenous Bregs in restraining excessive inflammation was demonstrated in mice lacking IL-10-producing B cells. In the absence of Bregs, mice developed an exacerbated arthritis (6). The exacerbation of disease was paralleled by an increase in T_h_1 and T_h_17 cells and a decrease in FoxP3^+^ Tregs, compared with wild-type (WT) mice (6).

Because of its crucial role in mediating Breg suppression, IL-10 production is largely used as a marker for Breg identification (1). To date, a number of Breg subsets with overlapping phenotypes and functions have been identified in various mouse models. Table 1 summarizes the different Breg subsets in the mouse that have been reported in the literature.

**Table 1.** Phenotypes of mouse Breg subsets

Subtype	Phenotype	Mechanism of suppression	References
T2-MZP B cells	CD19^+^CD21^hi^CD23^hi^CD24^hi^IgM^hi^IgD^hi^CD1d^hi^	IL-10	(7)
MZ B cells	CD19^+^CD21^hi^CD23^-^CD24^hi^IgM^hi^IgD^lo^CD1d^hi^	IL-10	(8)
B10 cells	CD19^hi^CD1d^hi^CD5^+^	IL-10	(9)
B-1a cells	CD5^+^	IL-10	(10)
Killer B cells	CD5^+^CD178^+^	FasL	(11)
GIFT-15 B cells	B220^+^CD21^+^CD22^+^CD23^+^CD24^+^ CD1d^+^CD138^+^IgD^+^IgM^+^	IL-10	(12)
Plasma cells	CD138^hi^IgM^+^TACI^+^CXCR4^+^CD1d^hi^Tim1^int^	IL-10, IL-35	(13)
Plasmablasts	CD138^+^CD44^hi^	IL-10	(14)
TIM-1^+^B cells	-	IL-10	(15)
PD-L1^hi^ B cells	CD19^+^PD-L1^hi^	PD-L1	(16)
—	B220^+^CD39^+^CD73^+^	Adenosine	(17)

### Transitional 2-marginal zone precursor cells

Our laboratory initially described the immunosuppressive functions of splenic transitional 2-marginal zone precursor (T2-MZP) B cells with a CD19^+^CD21^hi^CD23^hi^CD24^hi^IgM^hi^IgD^hi^CD1d^hi^ phenotype (7). Among the different B-cell subsets residing in the spleens of mice with arthritis, T2-MZP B cells were found to be the main producers of IL-10 after stimulation with collagen, and the only subset that displayed a suppressive capacity both *in vivo* and *in vitro* (7). The suppressive functions of IL-10-producing T2-MZP B cells have since been confirmed in a variety of immune-mediated pathologies ranging from autoimmune diseases to allergy and cancer (7,18–21). Adoptive transfer of T2-MZP Bregs has been reported to control the progression of arthritis. The mechanism of T2-MZP Breg-mediated suppression of arthritis included increased Treg and reduced T_h_1/T_h_17 frequencies, in both cases mediated by IL-10 (21). 

Unlike in arthritis, lupus-prone MRL-lpr mice lacked functionally suppressive T2-MZP Bregs (18). However, this defect was not permanent and was restored after *in vitro* stimulation of T2-MZP B cells with agonistic anti-CD40 mAb. Transfer of CD40-activated T2-MZP Bregs inhibited the development of lupus in recipient mice via the induction of IL-10-producing Tregs (18). T2-MZP Bregs have also been shown to suppress ovalbumin-induced allergic airway inflammation by inducing the infiltration of FoxP3^+^ Tregs into the sensitized lung following *Schistosoma mansoni* infection (20). Similarly, T2-MZP Bregs have been identified to suppress Helicobacter-induced gastric immunopathology by inducing IL-10-producing T-regulatory 1 (Tr1) cells (22). Furthermore, in a mouse model of transplantation, T2-MZP B cells from tolerized mice have been shown to prolong skin allograft survival by suppressing T-cell activation (23).

There are several other reports describing Breg subsets that share a partial T2-MZP Breg phenotype. Changes in the phenotype may be the result of adaptation of T2-MZP Bregs to the environment rather than existence of multiple Breg progenitors. For example, IL-10-producing CD19^+^CD21^hi^ B cells have been shown to repress antitumor immunity during squamous carcinogenesis (19). These cells express high levels of CD21 but not other markers, suggesting that other surface markers may have been down-regulated in response to specific stimuli present during the progression of cancer. IL-10-producing Bregs induced by a granulocyte macrophage colony-stimulating factor (GM-CSF)–IL-15 fusion protein, known as GIFT15-Bregs, also share many surface markers with T2-MZP Bregs including CD21, CD23, CD24, CD1d, IgD and IgM (12). However, GIFT15-Bregs have lost the expression of CD19 and gained CD138 expression, giving them a phenotype that is also similar to plasma cells. Adoptive transfer of GIFT15-Bregs suppressed the development of EAE via the production of IL-10 and by up-regulation of STAT-6 and MHC class II expression by GIFT15-Bregs (12).

In the spleen, along with T2-MZP B cells, their direct descendants—marginal zone (MZ) B cells—also express high levels of CD1d (24) and produce IL-10 and have been ascribed with regulatory properties. In response to Toll-like receptor (TLR) stimulation or apoptotic cells, MZ B cells produced the majority of IL-10 among B-cell subsets (8). Adoptive transfer of B cells stimulated with apoptotic cells protected mice from CIA via IL-10 release; however, the suppressive capacity of purified MZ B cells was not assessed (8). More recently, MZ B cells have been reported to suppress antigen-specific CD8^+^ T-cell responses during early stages of *Leishmania donovani* infection (25).

### B10 cells

Although MZ B cells express the highest levels of CD1d in the spleen, high expression of CD1d is a shared feature between different Breg subsets, both in mice and humans. However, the use of CD1d alone as a marker for the identification of Bregs is inadequate due to possible differences in gating strategies or exposure of B cells to different inflammatory environments. The co-expression of CD1d and CD5 has been used to characterize a population of splenic B cells, which produce exclusively IL-10, known as B10 cells (9). CD1d^hi^CD5^+^ B10 cells have been shown to suppress inflammation in a variety of immune-related disorders upon *ex vivo* stimulation with LPS, phorbal 12-myristate 13-acetate (PMA), ionomycin and monensin (L+PIM) (9, 26). Their relevance in modulating immune responses was firstly demonstrated in an EAE model, in which depletion of B cells prior to disease induction resulted in severely exacerbated disease and increased T-cell infiltration into the central nervous system. Additionally, adoptive transfer of splenic B10 cells also ameliorated EAE, particularly if administered at an early stage of disease (27). The efficacy of B10 cells in dampening autoimmunity has been shown in several experimental models including arthritis, lupus and intestinal inflammation (28–30). Similar to other Breg subsets, the induction of functionally suppressive B10 cells requires both cognate interactions with activated CD4^+^T cells expressing CD40L as well as soluble mediators including IL-21. B10 cells also require the expression of MHC-II, as MHC-II-deficient mice lack functional B10 cells (31). We refer the readers to the extensive review on the function of B10 cells in Reference (32).

### CD138^+^ B cells

Until recently, Breg subsets were thought to be splenic B cells at a stage of development preceding terminally differentiated plasma cells (1). However, new emerging evidence suggests that B cells at later stages of development also produce IL-10 and exhibit suppressive capacity. B10 cells were initially reported to differentiate into plasmablasts upon *in vitro* or *in vivo* activation; however, the regulatory capacity of the B10-cell-derived plasmablasts was not assessed (33).

Matsumoto *et al.* have recently shown that IL-10-producing CD138^+^ plasmablasts in the draining lymph nodes (dLNs) of mice with EAE are crucial in suppressing autoimmune inflammation by inhibition of dendritic cell function in pathogenic T-cell generation (14). They demonstrate that mice with B cells deficient in genes that control plasma cell differentiation, Prdm1 and IRF4, develop more severe EAE compared with control mice. Furthermore, their data suggest that the suppression of disease by Bregs in EAE does not require splenic B cells. This observation is in opposition to most Breg studies showing that Bregs reside in the spleen. In particular, adoptive transfer of splenic B10 cells has been shown to suppress colitis, and it was suggested that they suppress disease before developing into antibody-secreting cells (33). Whether B10 cells migrate to the dLNs and differentiate into plasmablasts to exert suppressive function *in vivo* remains to be investigated.

Unlike CD138^+^ plasmablasts in the LNs, splenic CD138^+^ B cells can suppress inflammation in EAE as well as immune response to *Salmonella* infection via the provision of cytokines IL-10 and IL-35 (13). IL-35 is an immunosuppressive heterodimeric cytokine comprising two IL-12 family subunits, p35 and EB13, and has been shown to be important for Treg-mediated suppression (34, 35). In addition to inducing Tregs, IL-35 has recently been shown to induce Bregs producing both IL-10 and IL-35, conferring protection against experimental autoimmune uveitis (EAU) (36). Adoptive transfer of IL-35-induced Bregs suppressed EAU by inhibiting T_h_1 and T_h_17 cells and promoting Tregs. It is noteworthy to mention that whereas in the above study, IL-35 was found to be important in the generation of IL-10-producing Bregs; Shen *et al*. showed that IL-35 is also required in the effector phase of Bregs. Indeed, chimeric mice with B cells lacking the expression of either IL-35 subunit (p35 or EB13) or IL-10 developed equivalently exacerbated EAE and improved resistance to *Salmonella typhimurium* infection, compared with WT mice, suggesting that both cytokines contribute equally to the effector function of Bregs (13). The discrepancy between the two studies could be the result of different experimental disease models or the different stimulations used to expand Bregs. Despite the discordance, both studies provide strong support for immunosuppressive functions of IL-35^+^ Bregs. Taken together, CD138^+^ B cells may represent distinct populations of Breg subsets, or the same Breg subset that adapts to different environments in the spleen and LNs, and consequentially releases different cytokines.

### TIM-1^+^ B cells

T-cell Ig mucin domain-1 (TIM-1) is a transmembrane glycoprotein that is associated with regulation of immune responses (37). TIM-1 is expressed by majority of IL-10-producing B cells in all major mouse Breg sub-populations, including T2-MZP B cells, B10 cells, CD138^+^ B cells and IgA^+^ plasmocytes (13, 15, 38). There is also emerging evidence identifying the regulatory capacity of TIM-1^+^ B cells in humans (39, 40). Furthermore, TIM-1 ligation in mice prolonged allograft survival by the induction of Bregs. The importance of TIM-1 in the maintenance and induction of IL-10-producing Bregs is highlighted by several independent studies. In one study, TIM-1 (mucin)^–/–^ mice showed impaired IL-10 production leading to spontaneous systemic autoimmunity in aged mice, associated with hyperactive T cells and increased levels of circulating antibodies (41). TIM-1^–/–^ mice have also been shown to spontaneously develop severe multiorgan tissue inflammation with age, due to defective IL-10 production by B cells, which was paralleled by an increase in pro-inflammatory cytokine production (42). Moreover, adoptive transfer of TIM-1^–/–^ B cells and WT T cells into Rag1^–/–^ mice immunized with myelin oligodendrocyte glycoprotein (MOG) peptide resulted in a more severe EAE compared with mice co-transferred with WT B cells and WT T cells. TIM-1^–/–^ B cells contributed to the severity of EAE by promoting increased T_h_1 and T_h_17 responses and by suppressing expansion of Tregs (42). In agreement with these data, another study has reported that TIM-1^–/–^ mice displayed accelerated graft rejection due to defects in both baseline and induced IL-10^+^ Bregs (43). Moreover, they demonstrated that a single transfer of WT TIM-1^+^ B cells to these mice could restore long-term graft survival. Of interest, both studies showed that whereas treatment with apoptotic cells expands IL-10-producing Bregs in WT mice, this does not occur in TIM-1^–/–^ mice (42, 43). Collectively, the data suggest that TIM-1 is critical in both the maintenance and induction of Bregs under physiological conditions.

### B-1a cells

While the majority of Breg subsets described are derived from ‘conventional’ B-2 cells, Bregs have also been identified within the B-1 lineage. B-1 cells are innate immune cells that produce the majority of natural antibodies, in particular IgM, to up-regulate the clearance of apoptotic cells (44). Because of its polyreactivity, natural IgM acts as a first line of defense against pathogens. B-1 cells can be further classified into functionally distinct subsets based on expression of CD5. CD5^+^ B-1 cells, known as B-1a cells, are major producers of IL-10 following innate activation (10).

B-1a cells were shown to be crucial in the control of TLR-mediated lethal inflammation in neonatal mice by dampening excessive inflammation in an IL-10-dependent manner (45). Further studies revealed that B-1a cells expressing Fas ligand (FasL), denoted as killer B cells, mediate CD4^+^ T-cell apoptosis during schistosomal infection, thereby preventing granulomatous inflammation (11). The maximal FasL expression on B-1a cells was observed upon stimulation with IL-10, IL-4 and soluble egg antigens (46). Interestingly, it has also been shown that protection against colitis in TCRα-deficient mice kept in a conventional environment, unlike those housed in pathogen-free conditions, was associated with expansion of IgM-producing B-1a cells (47). This was further supported by data showing that μMT/TCRα double-knockout mice kept in a conventional environment were not protected against colitis, suggesting that B-1a cells play a protective role in this model (47). Thus, innate B-1a Bregs could play an important role in immune regulation, as they not only neutralize invading pathogens but also suppress the inflammatory responses.

### Other Breg subsets

There is an ever-increasing list of new phenotypic and functional markers associated with Bregs. Independent of IL-10 production, PD-L1^hi^ B cells, interacting with CD4^+^CXCR5^+^PD-1^+^ T follicular helper (Tfh) cells, can limit both memory B-cell development and plasma cell differentiation. Amelioration of EAE on adoptive transfer of PD-L1^hi^ B cells to MOG-primed mice was associated with reduced Tfh expansion and generation of MOG-specific IgG. The significance of these results is intriguing considering that B cells do not play a pathogenic role in this model and that B-cell-deficient mice developed an exacerbated disease. Notably, PD-L1^hi^ B cells showed resistance to anti-CD20 therapy by sequestering B-cell activating factor from the milieu and promoting their survival (16). 

An elegant study using a mouse model of prostate cancer (PC) has identified IgA^+^ plasmocytes intratumorally, and shown that this B-cell subset can prevent oxaliplatin-mediated tumor-directed cytotoxic T-cell functions, via the expression of IL-10 and PD-L1 (38). Transforming growth factor (TGF)-β signaling and IgA class switch recombination were required for the development of this immune-suppressive Breg subset (38). Interestingly, IL-10-producing IgA^+^ plasmocytes were also identified in therapy-resistant and metastatic human PC, highlighting the potent immunosuppressive functions of this subset in cancer (38).

Additional mechanisms of immune suppression mediated by B cells include expression of CD39 and CD73, two ectoenzymes that together catalyze the dephosphorylation of adenine nucleotides to adenosine (17). Adenosine is known to suppress effector T-cell functions through binding to several adenosine receptors (48). Whereas CD39 expression is common to B cells, CD73 expression is not; B-1 cells and B10 cells display high expression of CD73, whereas fewer B-2 cells express CD73. Transfer of CD73^+^ B cells to CD73-deficient mice resulted in amelioration of severity of colitis, indicating that CD39^+^CD73^+^ B-cell adenosine can modulate colitis. An unexpected link between IL-10 and adenosine was observed in B-cell IL-10-deficient mice, wherein adenosine production was impaired due to reduced CD73 expression (17). Taken together, the current literature in mouse models highlights the diverse mechanisms of suppression employed by Bregs to limit excessive inflammation.

## Human Breg subsets

In healthy individuals, Bregs are important in the maintenance of immune homeostasis. Similar to mouse models, human Bregs are predominantly identified based on their ability to produce IL-10 (1). Patients with multiple sclerosis (MS) who were infected with helminths showed an increased frequency of IL-10-producing CD19^+^CD1d^hi^ B cells and, most importantly, a better clinical outcome. The B cells isolated from MS patients with helminth infection suppressed T-cell proliferation as well as their IFN-γ production (49). Although, in human studies, it is difficult to conclusively pin down the exact mechanism of action, it was suggested that the amelioration of disease symptoms in helminth-infected MS patients could be due to expansion of IL-10-producing B cells. To date, a number of reports describe different Breg subsets in humans and their mechanisms of suppression, summarized in Table 2.

**Table 2.** Phenotypes of human Breg subsets

Subtype	Phenotype	Mechanism of suppression	References
Immature B cells	CD19^+^CD24^hi^CD38^hi^	IL-10, PD-L1	(50)
B10 cells	CD19^+^CD24^hi^CD27^+^	IL-10	(51)
GrB^+^B cells	CD19^+^CD38^+^CD1d^+^IgM^+^CD147^+^	GrB, IL-10, IDO	(52)
Br1 cells	CD25^hi^CD71^hi^CD73^lo^	IL-10, IgG4	(53)
Plasmablasts	CD27^int^CD38^hi^	IL-10	(14)
—	CD39^+^CD73^+^	Adenosine	(54)
iBregs	—	TGF-β, IDO	(55)

### CD24^hi^CD38^hi^ Bregs

Bregs have been reported within the CD19^+^CD24^hi^CD38^hi^ immature B-cell population in peripheral blood of healthy individuals (50). Upon *in vitro* CD40 engagement, CD19^+^CD24^hi^CD38^hi^ B cells produced the highest amount of IL-10 among the B-cell subsets populating the peripheral blood and most importantly were the only subset to suppress T_h_1 differentiation. In addition to inhibiting T_h_1 responses and T_h_17 differentiation, CD19^+^CD24^hi^CD38^hi^ Bregs could also convert CD4^+^ T cells into Tregs and Tr1 cells (56). The inhibition of the T_h_1 response by Bregs was partially IL-10 dependent and required CD80–CD86 interaction with T cells (50).

Numerical and functional CD19^+^CD24^hi^CD38^hi^ Breg defects have been described in several autoimmune diseases, including systemic lupus erythematosus (SLE) and rheumatoid arthritis (RA) (50,56,57). CD19^+^CD24^hi^CD38^hi^ Bregs isolated from SLE patients show impaired IL-10 production upon activation via CD40, but not TLR9, and are unable to suppress T_h_1 responses due to a defect in STAT3 phosphorylation (50). It is hypothesized that the decreased IL-10 production might be a result of overstimulation of Bregs due to excessive inflammatory signals, leading to exhaustion and loss of suppressive function (1). In patients with active RA, Bregs were numerically impaired compared with healthy individuals and were unable to induce Tregs or suppress T_h_17 responses (56).

Studying the role of Bregs in SLE patients undergoing B-cell depletion (rituximab) therapy has provided useful insight into the contribution of Bregs to the maintenance of tolerance. In rituximab-treated patients, a higher immature to memory ratio has been associated with long-term remission (58, 59), suggesting that repopulation with immature Bregs might be associated with better disease outcome (1). This is supported by results in SLE patients treated with rituximab, where normalization of CD1d expression on newly repopulated CD19^+^CD24^hi^CD38^hi^ B cells corresponded to normalization of the invariant natural killer T (iNKT) cell number and function, as well as improved clinical response (57). iNKT cell number and function were otherwise impaired in SLE patients, due to defective B-cell-mediated stimulation, associated with altered CD1d recycling (57). These studies reveal that the newly repopulated B cells that have reacquired the capacity to differentiate into Bregs can condition the response of other immunoregulatory cells.

Although primarily studied in autoimmunity, CD19^+^CD24^hi^CD38^hi^ Bregs have been shown to participate in the immune response in infectious diseases and transplantation. In patients with HIV infection, enrichment in IL-10-producing CD19^+^CD24^hi^CD38^hi^ Bregs has been shown to correlate positively with the viral load (60). In the same studies, Bregs were shown to suppress HIV-1-specific CD8^+^ T-cell responses via IL-10 production and possibly PD-L1 expression. Moreover, depletion of Bregs from PBMCs resulted in enhanced CD8^+^ T-cell effector function as well clearance of infected CD4^+^ T cells *in vitro*. More recently, blockade of IL-10R and PD-1–PD-L1 interaction confirmed that Bregs inhibit antigen presentation, CD4^+^ T-cell proliferation and impaired anti-HIV cytotoxic T lymphocyte functions in HIV-infected patients (61). These results suggest that CD19^+^CD24^hi^CD38^hi^ Bregs may contribute to immune dysfunction in HIV infection and hinder viral eradication.

Recent studies from chronic graft-versus-host disease (cGVHD) have also highlighted the role of CD19^+^CD24^hi^CD38^hi^ Bregs in establishing transplant tolerance by suppressing effector T-cell responses (62, 63). Bregs within the CD19^+^CD24^hi^CD38^hi^ population as well as the CD19^+^IgM^+^CD27^+^ population displayed reduced IL-10-producing capacity in cGVHD patients, compared with patients without cGVHD and healthy donors (63). Furthermore, transplant-tolerant patients have been reported to display significantly higher IL-10-producing CD19^+^CD24^hi^CD38^hi^ Bregs than immunosuppressed patients, but not healthy controls (64). More recently, it has been shown that the ratio of IL-10/TNF-α expression by CD19^+^CD24^hi^CD38^hi^ B cells as a measure of cytokine polarization is a better indicator of Breg function than IL-10 expression alone. Although renal transplant patients both with stable graft function as well as graft rejection displayed similar IL-10 expression levels by CD19^+^CD24^hi^CD38^hi^ B cells, the IL-10/TNF-α ratio was lower in patients who subsequently displayed graft dysfunction (65). A recent prospective study in kidney transplant patients confirmed the importance of Bregs in transplant tolerance, as CD19^+^CD24^hi^CD38^hi^ B-cell numbers were found to be associated with reduced rejection rates (66). Thus, these studies support a critical role for Bregs as a biomarker and for therapeutic intervention in transplantation tolerance.

A recent study suggests the existence of immature B-cell-derived regulatory plasmablasts in humans (14). Stimulation of naive immature B cells with CpG, IFN-α, IL-6 and IL-2 resulted in the maximal expansion of IL-10-producing CD27^int^CD38^hi^ plasmablast-like cells with up-regulation of IRF4, Blimp1 and XBP1 expression. While this suggests a developmental link between immature Bregs and IL-10-producing plasmablasts, further studies will be necessary to determine whether immature B cells are really the precursors of regulatory plasmablasts. It remains to be ascertained whether in addition to producing IL-10, plasmablast-like B cells are also functionally suppressive. 

### B10 cells

A population of IL-10-producing Bregs named B10 cells has recently been described in peripheral blood. The majority of the B10 cells were found within the CD19^+^CD24^hi^CD27^+^ B-cell sub-population, which via the production of IL-10 suppressed TNF-α production by monocytes. Unlike CD40-activated CD24^hi^CD38^hi^ Bregs, human B10 cells respond to stimulation with LPS and CpG (51). The frequency of B10 cells was reportedly increased in several systemic and organ-specific autoimmune diseases (51). We refer the readers to a fully comprehensive review on the role of B10 cells in health and disease in Reference 32. 

### Other Breg subsets

Recent advances in our understanding of human Breg functions have revealed an important role for Bregs in tumor immunology. A study has shown that granzyme B (GrB)-expressing B cells with a CD19^+^CD38^+^CD1d^+^IgM^+^CD147^+^ phenotype infiltrate tumors and regulate T-cell responses (52). GrB^+^ Bregs that were induced by IL-21 production by T cells also expressed IL-10, indoleamine-2,3-dioxygenase (IDO) and CD25 (52). More detailed experiments are required to better understand the role of GrB^+^ Bregs and to exploit their therapeutic potential.

Another subset of IL-10^+^ Bregs, known as B regulatory 1 (Br1) cells, displays a CD25^hi^CD71^hi^CD73^lo^ phenotype (53). These cells play an important role in allergen tolerance by suppressing antigen-specific CD4^+^ T-cell proliferation as well as by producing anti-inflammatory allergen-specific IgG4 antibodies and contributing to peripheral tolerance (53).

Independent of IL-10, circulating CD39^+^CD73^+^ B cells can drive a shift from an ATP-driven pro-inflammatory environment to an anti-inflammatory milieu induced by adenosine (54). As described above, CD39 and CD73 are ectonucleotidases that hydrolyze exogenous ATP to adenosine 5′-monophosphate (5′-AMP) and adenosine. While autocrine adenosine regulates B-cell responses, *in vitro* activated B cells down-regulated CD73 and inhibited CD4^+^ and CD8^+^ T-cell proliferation, possibly via production of 5′-AMP (54). The role of this novel mechanism of immune suppression by CD39^+^CD73^+^ B cells in health and disease requires further investigation.

Another mechanism of Breg suppression involves the production of TGF-β and IDO and induces the expansion of IL-10^+^ and TGF-β^+^ Tregs (55). The expansion of these induced Bregs (iBregs) requires stimulation with CTLA-4 by T cells. Collectively, the emerging roles of human Bregs in maintaining homeostasis emphasize the importance of this population in restraining inflammatory responses.

## Does a Breg lineage marker exist?

The multiple distinct, yet overlapping Breg phenotypes highlight the need for more extensive characterization of both mouse and human Bregs to improve the current understanding of Breg biology. Although IL-10 is still regarded as being crucial to Breg function, the identification of other diverse suppressive mechanisms raises several new possibilities. It remains to be seen whether functions are strictly restricted based on Breg phenotype. For these reasons, *in vitro* or *in vivo* suppression currently remains the gold standard for Breg identification (1).

**Fig. 1.**
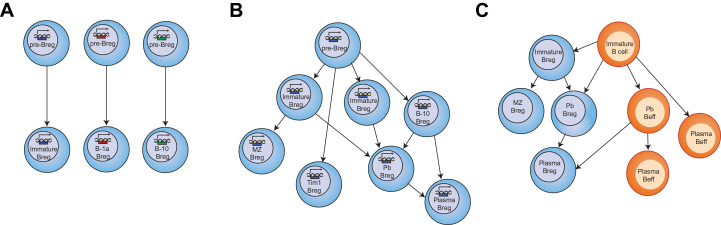
Models of Breg development. Several models could explain the existence of numerous Breg subsets. (**A**) Multi-lineage Bregs. In this model, different Breg subsets arise from individual progenitors. (**B**) Single lineage Bregs. All the different Bregs subsets described (with the exception of B1) arise from a single progenitor and express a single transcription factor. Bregs are not a terminally differentiated state and can exhibit suppressive functions at different stages of differentiation. (**C**) Induced Bregs. In this model, any B cell can become regulatory on exposure to specific environmental stimuli and exhibit suppressive capacity. Beff, B effector cell; Pb, plasmablast; Plasma, plasma cell.

The question of whether Bregs represent a dedicated functional lineage or whether they represent multiple lineages is yet to be answered. Although the existence of a Treg-FoxP3-equivalent transcription factor remains unknown, the possibility of a developmental relationship between the subsets cannot be excluded. Different Breg subsets could either arise from individual progenitors or could share a common progenitor. For instance, CD5, CD1d, CD21 and TIM-1 expression alone or in combination have been shown to ‘capture’ the majority of IL-10-producing Bregs in mice (13, 15, 38). It is possible that these markers identify Bregs at an early stage of differentiation but that their phenotype changes in response to the environment in which they reside. Another possibility is that the induction of Bregs is simply a consequence of the environmental milieu and that any B cell can acquire regulatory functions. This hypothesis is supported by the findings showing that stimulation with different combinations of cytokines induces different Breg subsets (31, 36, 67, 68). The proposed models of Breg development are summarized in Fig. 1.

The shared surface markers and mechanisms of suppression between different Breg subsets both in mice and humans raise several interesting questions. Do the increased Bregs in experimental manipulations expand a preexisting population of Bregs? Moreover, if any B cell can acquire a Breg phenotype, is this a transient state or a state of terminal differentiation? It appears that, based on the immunological requirements, any B cell can acquire either effector or regulatory functions, or simultaneously exhibit both functions as seen with regulatory plasmablasts and Br1 cells (14, 53).

Recently published data suggest that B cells at different stages of development, from immature cells to terminally differentiated plasma cells, can acquire suppressive functions indicating that Bregs are not a terminally differentiated state (13, 14). For these reasons, B-cell depletion therapies must be carefully designed as elimination of all B cells or certain B-cell subsets can result in an unexpected clinical response. Improved knowledge of Breg subsets, their role in different stages of disease and how they are induced can provide new opportunities to develop novel therapies targeting Bregs. Therapies inducing Bregs *in vivo* or stimulating them *in vitro* by modulating the environmental milieu could provide several advantages over the currently used B-cell-depletion therapies.

## Conclusion

The past decade has provided striking new insights into the diverse phenotypic and functional subsets of Bregs. The different diseases resulting from disruption of Breg homeostasis emphasize the importance of immunosuppressive Bregs. Further investigation into Breg biology and the signals that drive Breg differentiation could provide ways of reshaping and resetting the immune system for better treatment of various immune-related pathologies.
